# Association between Secreted Phosphoprotein-1 (*SPP1*) Polymorphisms and Low Bone Mineral Density in Women

**DOI:** 10.1371/journal.pone.0097428

**Published:** 2014-05-15

**Authors:** Jen-Hau Chen, Yen-Ching Chen, Chien-Lin Mao, Jeng-Min Chiou, Chwen Keng Tsao, Keh-Sung Tsai

**Affiliations:** 1 Department of Geriatrics and Gerontology, National Taiwan University Hospital, No. 1, Taipei, Taiwan; 2 Department of Internal Medicine, National Taiwan University Hospital, No. 7, Taipei, Taiwan; 3 Institute of Epidemiology and Preventive Medicine, College of Public Health, National Taiwan University, Taipei, Taiwan; 4 Institute of Statistical Science, Academia Sinica, Nankang, Taipei, Taiwan; 5 MJ Health Management Institution, 12F., No. 413, Section 4, Taipei, Taiwan; 6 Department of Laboratory Medicine, National Taiwan University Hospital, No. 7, Taipei, Taiwan,; CAEBi, Spain

## Abstract

**Background:**

A recent meta-analysis found that secreted phosphoprotein-1 (SPP1) can predict the risk of both osteoporosis and fracture. No study has explored the association of *SPP1* haplotype-tagging single nucleotide polymorphisms (htSNPs) and haplotypes with bone mineral density (BMD).

**Methods:**

This is a cross-sectional study. A total of 1,313 healthy Taiwanese women aged 40 to 55 years were recruited from MJ Health Management Institute from 2009 to 2010. BMD was dichotomized into high and low BMD groups. Three common (allele frequency ≥5%) htSNPs were selected to examine the association between sequence variants of *SPP1* and BMD.

**Results:**

Homozygosity for the T allele of rs4754 were protective from low BMD [TT vs. CC: adjusted OR (AOR)  = 0.58, 95% confidence interval (CI)  = 0.83–0.89]. A protective effect was also found for women carrying 2 copies of Hap3 TCT (AOR  = 0.57, 95% CI  = 0.34–0.95). Menopausal status marginally interacted with *SPP1* rs6839524 on BMD (*p* = 0.049). Postmenopausal women carrying variant rs6839524 (GG+GC vs. CC: AOR  = 2.35, 95% CI  = 1.06–5.20) or Hap1 TGC (AOR  = 2.36, 95% CI  = 1.06–5.24) were associated with 2.4-fold risk of low BMD. For women with low BMI (<18.5 kg/m^2^), variant rs6839524 (AOR  = 7.64) and Hap1 (AOR  = 6.42) were associated with increased risk of low BMD. These findings did not reach statistical significance after correction for multiple tests.

**Conclusions:**

*SPP1* htSNP protected against low BMD in middle-aged women. *SPP1* genetic markers may be important for the prediction of osteoporosis at an early age.

## Introduction

Osteoporosis, characterized by low bone mass and propensity to fracture, has become a global health issue as the rapid growth of aging population [1], [2]. About 21% of women aged 50 to 84 years has osteoporosis, which is three times higher than that in men [3]. In the US (1988–1994), the prevalence of osteoporosis was highest among white (19%), followed by Mexican American (16%) and black (7%) [4], which in part correspondence to the levels of bone mineral density (BMD) ranged from lowest in Asian, followed by native Americans, Hispanic, and then African American [5]. This may be explained by different lifestyle in the East and West. Compared with other Asian countries, Japanese and Korean, but not Taiwanese, women showed clear age-dependent loss of BMD [6]. Osteoporosis is a “silent disease” until a sudden strain, twist, fall, or fracture, which is associated with increased mortality in later life [1]. Therefore, it is important to identify osteoporosis risk at an early age to prevent fall and fracture risk in late life.

Secreted phosphoprotein-1 (SPP1) is known as osteopontin. It is a glycoprotein related to bone formation and anchoring of osteoclasts to the bone remodeling matrix via binding with vitronectin receptor [7], [8]. SPP1 exists in osteoblasts and mineralized bone matrix and intramembranous ossification [7], which enhances osteoblastic differentiation and proliferation [9], [10]. SPP1 modulates both bone formation and resorption [11]. As compared to wildtype mice, *SPP1* gene knockout mice are resistant to bone resorption [12]. A Chinese study found that the heritability of BMD was quite high (0.6 to 0.9, vary by body site and sex) [13], therefore, genetic difference may pay a role on BMD level. Because SPP1 is involved in osteogenesis and bone remodeling, *SPP1* polymorphisms may play an important role in the pathogenesis of osteoporosis. Variation on functional single nucleotide polymorphisms (SNPs) may affect the production of *SPP1* protein and then bone formation; intronic SNPs may affect BMD via regulating the alternative splicing and the subsequent protein production [14], [15].

Few epidemiologic studies have explored the association between *SPP1* polymorphisms and osteoporosis or BMD. One study found that increased plasma SPP1 level was associated with low BMD [16]. The other candidate-gene study including white and African Americans reported no difference between average hip or spine BMD level by genotypes of *SPP1* rs11730582 or rs4754 [17]. However, the selection of these two SNPs only captured limited genetic information in *SPP1* gene (r^2^ = 0.62). In addition, these studies did not assess the association between *SPP1* genetic polymorphisms and low BMD or osteoporosis. A meta-analysis study, included 5 genome-wide association studies [GWASs, using data mining approach to explore a massive number of SNPs for specific outcome(s)] on BMD and fracture, found that polymorphisms of 9 genes (*ESR1*, *LRP4*, *ITGA1*, *LRP5*, *SOST*, *SPP1*, *TNFRSF11A*, *TNFRSF11B*, and *TNFSF11*) were significantly associated with BMD level; and *SPP1*, *SOST*, *LRP5*, and *TNFRSF11A* were also related to elevated risk of fracture [18]. Another recent meta-analysis, included 17 GWASs on BMD, found that polymorphisms of *FAM210A, SLC25A13, LRP5, MEPE*/*SPP1*, *SPTBN1*, and *DKK1* were significantly associated with both BMD and fracture risk [19]. Among the genes that can predict both BMD and fracture risk, *SPP1* is the only one that modulates both osteoblast and bone resorption [8], and associated with the risk of both vertebral and non-vertebral fracture [18]. In addition, no studies have explored how *SPP1* genetic polymorphisms affect the risk of low BMD by using representative haplotype-tagging single nucleotide polymorphisms (htSNPs) and haplotypes. Data in Asian is also lacking.

Because of the above research gap, this study was aimed to explore the association between *SPP1* polymorphisms and the risk of low BMD in middle-aged women. A systematic approach was used to select representative htSNPs in *SPP1* to capture sufficient genetic information and to identify SNPs representative for Asian population. We also evaluated the interactions between *SPP1* polymorphisms and menopausal status or body mass index (BMI) on BMD, which has not been explored previously.

## Materials and Methods

### Study population

This is a cross-sectional study. A total of 1,567 healthy Taiwanese (Chinese ethnicity) women, aged 40 to 55 years, were recruited from MJ Health Management Institution from October 2009 to August 2010. The outcome of this study is spinal BMD (g/cm^2^). Spinal BMD is the major site measured at the MJ Health Management Institute. Participants with the following conditions or diseases were excluded (n = 254): (1) diseases known to affect BMD levels (e.g., hyperparathyroidism, hyperthyroidism, type 1 diabetes, inflammatory bowel disease, chronic active hepatitis, liver cirrhosis, chronic cholestatic diseases, and multiple myeloma), (2) took medications for osteoporosis (e.g., raloxifene), (3) received hormone replacement therapy or other medications (e.g., steroid, oral contraceptive agents) that may affect BMD, (4) lack of BMD at lumbar spine, (5) lack of blood samples or genotyping data. A total of 1,313 women were included for data analyses.

A questionnaire was administered to collect information on demography, lifestyle (e.g., smoking status, alcohol consumption, and exercise), and disease history, etc. A blood sample was collected in an 8 ml EDTA tube from each participant. Genomic DNA was extracted by using QuickGene-Mini80 kit (Fujifilm, Tokyo, Japan). Participants with the following conditions were excluded: BMD was measured at sites other than spine (n = 85), lack of blood sample (n = 113), or genotyping data (n = 2), and had steroid or hormone therapy (n = 70). Some participants may lack of two or more information above.

### Ethics Statement

The study protocol has been approved by the Institutional Review Boards of National Taiwan University and MJ Health Management Institution. Written informed consent was obtained from each study participant. The consent from the legal guardian/next of kin was obtained when patients had serious cognitive impairment. This research carried out with human subjects complies with the World Medical Association Declaration of Helsinki - Ethical Principles for Medical Research Involving Human Subjects.

### Measurement of bone mineral density

The BMD (g/cm^2^) of the lumbar spine (L1-L4) was measured by a dual-energy X-ray absorptiometry densitometer (DXA, General Electric Lunar Health Care, DPX-L, USA). Calibration of BMD measurement was performed daily. The long-term coefficient of variation in BMD was around 1%. This healthy population included few participants with osteoporosis (<1%). Instead of using osteoporosis as the outcome variable, BMD was tertiled (i.e. T1, T2, and T3) on the basis of the data of the whole population in order to identify the subgroup with an elevated risk of low BMD. Previous studies [20], [21] and our recent study [22] have used similar approaches that involve BMD tertiles. The high BMD group comprised participants in T2 plus T3 (i.e., the reference group) and the low BMD group comprised participants in T1 (i.e., the comparison group) with a BMD cut-off point of 1.27 g/cm^2^.

### SNP selection and genotyping assays

Common (frequency ≥0.05) SNPs in *SPP1* were identified from genotyping data of Han Chinese in Beijing, China (CHB) of the International HapMap Project (http://hapmap.ncbi.nlm.nih.gov). Haplotype block was defined by Haploview program (http://www.broadinstitute.org/haploview/haploview) using modified Gabriel algorithm [23], [24]. Three representative htSNPs [rs11730582 in 5′untranslated region (UTR), rs6839524 in intron, and rs4754 in exon] were selected from 12 common SNPs using tagSNP program [25] based on the common disease/common variant hypothesis [26]. TaqMan Assay was used to determine genotypes using HT7900 (Applied Biosystems Inc., CA, USA). Genotyping success rate was greater than 95% for each SNP. Quality control samples were replicates of 5% study participants and the concordance rate was 100%.

### Statistical analyses

Hardy-Weinberg equilibrium (HWE) test was performed for each SNP to check genotyping error. The expectation-maximization algorithm was used to estimate haplotype frequencies in the haplotype block using tagSNP program [25]. High and low BMD was defined as above. Power analysis was performed by using QUANTO program (http://hydra.usc.edu/GxE/).

Logistic regression model was used to estimate the adjusted odds ratio (AOR) and 95% confidence interval (CI) for the risk of low BMD in participants carrying either 1 or 2 versus 0 copies of minor allele of each SNP and each multilocus haplotype. Haplotype trend regression [27] was used to test the global association between *SPP1* haplotypes and low BMD. Given a significant global test, haplotype-specific tests can provide some guidance as to which variant(s) contributes to the significant global test. The association between *SPP1* genetic polymorphisms and continuous BMD were also assessed by using general linear model (GLM). After stepwise model selection and the inclusion of variables with biological importance, age, menopausal status (yes/no), BMI (kg/m^2^), alkaline phosphatase (ALP, IU/L), uric acid (UA, mg/dL), low-density lipoprotein (LDL, mg/dL), and exercise (frequency × duration × intensity) were adjusted in the models. All participants had normal creatinine level (<1.3 mg/dL) and thus this variable was not explored in this study.

A likelihood ratio test was used to evaluate how menopausal status (pre- and post-menopause) and BMI groups (<18.5, 18.5 to <24, ≥24 kg/m^2^) modified the association between *SPP1* polymorphisms and risk of low BMD. Stratified analyses were performed by menopausal status and BMI groups. Correction for multiple tests was performed by false discovery rate (FDR) using method of Benjamini and Hochberg (1995) [28]. Statistical analyses were performed by using SAS 9.2 (SAS Institute, Cary, NC) and all statistical tests were two-sided.

## Results

### Characteristics of the study population

This study included 1,313 participants. The differences between participants with low and high BMD are summarized in Table 1

**Table 1:** Characteristics of the study population.

	Low BMD		High BMD	
Variables	(<1.27 g/cm^2^)		(≥1.27 g/cm^2^)	*p*
	n = 881		n = 432	
		**Mean ± SE**		
Age	46.8±0.2		45.6±0.2	**<0.001**
Alkaline phosphatase (IU/L)	61.5±0.6		55.5±0.7	**<0.001**
		**n (%)**		
Menopause				**<0.001**
Yes	227 (26)		45 (11)	
No	647 (74)		381 (89)	
Cigarette smoking				0.45
Yes	63 (8)		39 (9)	
No	777 (92)		378 (91)	
Alcohol consumption				0.84
Yes	50 (6)		27 (7)	
No	775 (94)		379 (93)	
Body mass index (kg/m^2^)				**<0.0001**
<18.5	56 (6)		15 (3)	
≥18.5 to <24	652 (74)		279 (65)	
≥24	172 (20)		137 (32)	
High-density lipoprotein (mg/dL)				0.71
≥50	808 (92)		396 (92)	
<50	73 (8)		33 (8)	
Low-density lipoprotein (mg/dL)				**0.0001**
<130	670 (76)		366 (85)	
≥130	211 (24)		63 (15)	
Triglyceride (mg/dL)				0.28
<150	794 (90)		381 (88)	
≥150	87 (10)		51 (12)	
Uric acid (mg/dL)				**0.006**
<6	806 (91)		374 (87)	
≥6	75 (9)		58 (13)	
Hypertension				0.48
Yes	88 (10)		49 (11)	
No	793 (90)		382 (89)	
Diabetes				0.05
Yes	262 (30)		151 (35)	
No	619 (70)		281 (65)	
Regular exercise				0.39
Yes	368 (47)		194 (50)	
No	412 (53)		195 (50)	

**Abbreviations:** BMD, bone mineral density; BMI, body mass index; hypertension, systolic blood pressure >140 mmHg or diastolic blood pressure >90 mmHg or had medication for controlling blood pressure; diabetes, fasting glucose ≥126 mg/dl or using medication for diabetes; regular exercise: walking or hiking ≥30 mins/2 to 3 days.

Numbers in bold indicate significant findings (*p*<0.05).

### *SPP1* polymorphisms and BMD

Three *SPP1* htSNPs [rs11730582 (5′ UTR), rs6829524 (intron), and rs4754 (exon)] were genotyped. The minor allele frequencies (MAFs) of these SNPs ranged from 31% to 42%, which were similar to the MAFs of CHB data from International HapMap Project (29 to 38%). All *SPP1* SNPs were in HWE among participants with low BMD, high BMD, or the whole population. Power analysis showed that given 881 and 432 participants with low and high BMD, respectively, rs4754 (MAF  = 0.31) has over 0.99 of power to detect an OR at 0.58. Because of the modest effect and high MAF, the power is low (<0.7) for rs11730582 (MAF  = 0.33) and rs6829524 (MAF  = 0.42) to detect an OR at 1.14 and 0.91, respectively.

Homozygosity for the T allele of rs4754 were protective from low BMD (TT vs. CC: AOR  = 0.58, 95% CI  = 0.83–0.89, *p* = 0.005, Table 2). This association did not reach statistical significance after correction for multiple tests. The other two htSNPs did not show significant relationship with the outcome.

**Table 2:** Association of *SPP1* common htSNPs and haplotypes with low BMD.

				Codominant Model					
	Freq.	0 copies		1 copy			2 copies		
	(%)	BMD (L/H)	AOR	BMD (L/H)	AOR (95% CI)	*p*	BMD (L/H)	AOR (95% CI)	*p*
SNP									
rs11730582		394/206	1.00	385/177	1.17 (0.89–1.53)	0.54	102/49	1.13 (0.73–1.74)	0.83
rs6839524		295/157	1.00	429/197	1.25 (0.94–1.67)	0.05	158/78	0.91 (0.63–1.32)	0.22
rs4754		439/195	1.00	368/179	1.07 (0.81–1.41)	0.02	74/57	**0.58 (0.83**–**0.89)**	**0.005**
**Haplotype (Global test ** ***P*** **<0.0001)**									
Hap1 TGC	41.2	299/162	1.00	431/193	1.30 (0.97–1.73)	0.08	151/77	0.90 (0.62–1.31)	0.58
Hap2 CCC	26.3	466/248	1.00	351/160	1.18 (0.90–1.55)	0.24	64/24	1.45 (0.83–2.53)	0.20
Hap3 TCT	23.9	522/243	1.00	317/153	1.08 (0.82–1.43)	0.60	42/36	**0.57 (0.34**–**0.95)**	**0.03**
Hap4 CCT	6.5	782/371	1.00	92/59	0.73 (0.49–1.09)	0.13	7/2	1.81 (0.35–9.34)	0.48
Total	97.9								

**Abbreviations:** SNP, single nucleotide polymorphism; Freq., haplotype frequency; BMD, bone mineral density; AOR, adjusted odds ratio; CI, confidence interval; L, low BMD; H, high BMD.

All models were adjusted for age, menopausal status, BMI (kg/m^2^), serum ALP (IU/L), UA (mg/dL), LDL (mg/dL), and exercise (frequency × duration × intensity).

The SNPs with underscore indicate variant allele.

Numbers in bold indicated significant findings (*p*<0.05).

Three common htSNPs (rs11730582, rs6839524, and rs4754) spanning *SPP1* gene formed one block using the modified Gabriel algorithm [23], [24]. Four common (frequency ≥0.05) haplotypes were identified (cumulative frequency, 97.9%); the global test for the association between *SPP1* haplotypes and low BMD was significant (*p*<0.0001, Table 2). Two copies of Hap3 TCT were protective from low BMD (AOR  = 0.57, 95% CI  = 0.34–0.95, *p* = 0.03, Table 2). Other haplotypes were not associated with the outcome. The conditional haplotype analysis was performed conditioning on other haplotypes and the results did not reach statistical significance (Hap1: ref; Hap2: AOR  = 1.14, 95% CI  = 0.90–1.44; Hap3: AOR  = 0.93, 95% CI  = 0.74–1.17; Hap4: AOR  = 0.85, 95% CI  = 0.59–1.24). These findings did not remain significant after correction for multiple tests.

We also kept only participants with the lowest and the highest BMD tertile and compared them for the same analyses above. Because of removing one-third of the study population (the 2^nd^ tertile), the statistical power decreased and the protective effect for rs4754 and Hap3 no longer reached statistical significance.

### Interactions between menopausal status and *SPP1* polymorphisms

Menopausal status has known as an important modifier for BMD. Interaction between menopausal status and *SPP1* htSNPs or haplotypes for low BMD did not reach statistical significance. After stratification by menopausal status, postmenopausal women carrying variant rs6839524 were associated with low BMD (GG+GC vs. CC: AOR  = 2.35, 95% CI  = 1.06–5.20, *p* = 0.03). Postmenopausal women carrying Hap1 TGC was associated with low BMD (AOR  = 2.36, 95% CI  = 1.06–5.24, *p* = 0.03). These findings did not reach statistical significance after correction for multiple tests. No significant association was found in other subgroups or for other *SPP1* htSNPs/haplotypes after stratification by menopausal status.

### Interaction between BMI and *SPP1* polymorphisms

No significant interaction was observed between *SPP1* SNPs or haplotypes and low BMD. After stratification by BMI groups (<18.5, 18.5 to <24, ≥24 kg/m^2^), women with low BMI (<18.5 kg/m^2^) carrying rs6839524 variant were associated with low BMD (GG+GC vs. CC: AOR  = 7.64, 95% CI  = 1.42–40.97, *p* = 0.02). Women with low BMI (<18.5 kg/m^2^) carrying Hap1 TGC were associated with low BMD (AOR  = 6.42, 95% CI = 1.23–33.60, *p* = 0.03). These findings did not reach statistical significance after correction for multiple tests.

The power for assessing interaction between *SPP1* SNPs and menopausal status or BMI is low because of the smaller sample size in subgroup analysis and the results should be interpreted with caution.

## Discussion

To the best of our knowledge, this is the first study exploring the association between *SPP1* polymorphisms and low BMD using htSNPs in Asian population. We found that homozygosity for the T allele of *SPP1* rs4754 (TT) and Hap3 TCT were associated with low BMD; the former result remained significant after correction for multiple tests for SNP analysis but lost significance after correction for multiple tests for SNP and haplotype analysis. The only candidate-gene study [17], which included white and black populations, only compared mean BMD by *SPP1* genotypes of 2 SNPs (i.e., no estimation of multivariable OR and 95% CI) and no significant difference was observed. Previous GWASs and meta-analysis [18], [19], [29]–[33] for BMD or fracture risk mainly focused on white population. In addition, haplotype analysis, which offers more information than single-locus SNPs, has not been performed previously. Therefore, our results provide important information because of the estimation of outcome risk by using multivariable OR, large sample size (n>1,300), selection of representative htSNP, and performing haplotype analysis.

Among 3 htSNPs genotyped in this study, rs4754 is a synonymous SNP. That is, rs4754 does not lead to the change of amino acid but may affect BMD level via its influence on translational efficiency. Interestingly, C allele is the major allele of rs4754 in Chinese (MAF: C = 0.66, T = 0.34) but the minor allele in white (MAF: C = 0.23, T = 0.77, http://hapmap.ncbi.nlm.nih.gov). Therefore, the inconsistent findings were observed between this Asian study and previous studies focused on whites [18], [19], [29]–[33]. Hap3 TCT also showed significant association with high BMD, which may be attributable to the only SNP rs4754 with the minor allele in Hap3. Three htSNPs were in one haplotype block and strong linkage disequilibrium (LD) were observed between rs11730582 and rs6839524 (|D′|  = 0.98) as well as between rs6839524 and rs4754 (|D′|  = 0.96). However, the pairwise r^2^ for any two SNPs were low (0.02 to 0.34). Especially, both D′ (0.30) and r^2^ (0.02) were low between rs11730582 and rs4754, this may explain the non-significant association between *SPP1* rs11730582 and BMD.

SPP1 plays a role in a wide spectrum of physiologic and pathologic processes [34]–[37]. First, it mediates the attachment of osteoclasts to bone matrix and then regulates bone resorption and normal bone development [37]. The polymorphisms of *SPP1* gene may regulate SPP1 structure, decrease serum SPP1 level, change or reduce SPP1 protein, which may affect bone formation, resorption and the osteoclastic process. The downgrading of osteoclastic process may slow BMD decline and thus prevent osteoporosis. Second, SPP1 plays an important role in regulating immune response [38]. Therefore, polymorphisms of *SPP1* may block or reduce the inflammation responses and thus showed increased BMD. In addition, *SPP1* polymorphisms may also interact with two important modifiers, menopausal status or BMI, on BMD as detailed below.

It has been known that sex hormone plays an important role in maintaining bone strength [39]. For most of women, BMD decreases rapidly during the first few years after menopause [40] as a result of excessive osteoclastic activities via unopposed osteoclastic activation after rapid declination of estrogen. Because SPP1 modulates osteoclast and thus sequence variants of *SPP1* may affect bone resorption. This may explain our finding that postmenopausal women carrying 1 or 2 copies of variant rs6839524 were associated with low BMD (AOR  = 2.35), which did not reach statistical significance after correction of multiple tests. An association was also observed for Hap1, which rs6839524 is the only SNP with minor allele. It is possible that variant rs6839524 affects bone formation and this effect becomes more evident after menopause. In addition, rs6839524 is an intronic SNP and its variation may affect the alternative splicing, e.g., altering mRNA folding or the stability of mRNA structure, and then the subsequent protein production [14], [15]. All these may explain the associations, which did not reach statistical significance after correction of multiple tests, between rs6839524 or Hap1 and low BMD in postmenopausal women.

BMI has been related to BMD previously. Low BMI has been associated with increased risk of osteoporosis and fracture [41] and the association varied by ethnic groups, e.g., positive association between one unit increase of BMI and BMD in white women but negative association was observed in African American women [42]. Because of different diet and lifestyle, body shape can be quite different between people in Western and Eastern countries. However, relevant data and research in Asian population are sparse. Our research, for the first time, explored that among women with low BMI (<18.5 kg/m^2^), variant carriers of rs6839524 (AOR  = 7.64) or Hap1 TGC (AOR  = 6.42) were associated with low BMD, which did not reach statistical significance after correction for multiple tests. The application of these polymorphisms will help us to identify women with low BMD.

This study has several strengths. First, the selections of a set of representative htSNPs for Asian captured the majority of genetic information of *SPP1* (r^2^ = 0.82, estimated by tagSNP program) as compared with that (r^2^ = 0.65) of the only candidate-gene study [17]. Second, haplotypes capture unknown variants via LD between these SNPs and thus provide more information than single SNP. In addition, unlike most previous studies, this study included premenopausal women (n∼1000) that allowed us to assess how menopausal status interacted with *SPP1* polymorphisms on BMD and, importantly, to predict outcome risk at an early age.

This study had some limitations. First, this is a cross-sectional study, which causal inference is usually not available. Functional analysis will be needed to unravel the underlying mechanism between *SPP1* polymorphisms and BMD. In addition, the original questionnaire did not collect fracture information. Because this population is healthy, fracture frequency is low. We also assessed the association between *SPP1* genetic polymorphisms and continuous BMD by using GLM and no significant findings were observed. Because the aim of this study is to identify a high-risk population of low BMD, no further analyses were performed by using continuous BMD as outcome.

*SPP1* plays a role in bone formation and resorption. This study has some first findings. Homozygosity for the T allele of rs4754 and Hap3 TCT in *SPP1* were significantly associated with low BMD in this Asian population. rs6839524 and haplotypes in *SPP1* have not been explored before. Variant carriers of rs6839524 and Hap 1 TGC were associated with low BMD in menopausal women or women with low BMI. These findings did not reach statistical significance after correction for multiple tests. Because of the complex role of *SPP1* in bone physiology, functional and larger studies are warranted to confirm our findings.
